# Natural and crystal bundles disaggregated palygorskite in young broilers: a comparison study

**DOI:** 10.5713/ab.25.0150

**Published:** 2025-08-12

**Authors:** Zichao Tan, Yueping Chen, Chao Wen, Aiqin Wang, Yanmin Zhou

**Affiliations:** 1 College of Animal Science and Technology, Nanjing Agricultural University, Nanjing, China; 2 Key Laboratory of Clay Mineral Applied Research of Gansu Province, Center of Eco-material and Green Chemistry, Lanzhou Institute of Chemical Physics, Chinese Academy of Sciences, Lanzhou, China

**Keywords:** Broiler, Digestive Function, Growth Performance, Intestinal Health, Palygorskite

## Abstract

**Objective:**

This study aimed to compare the effects of natural palygorskite (Nat-Pal) and crystal bundles disaggregated palygorskite (Dis-Pal) supplementation on the growth performance, intestinal health, and digestive function of young broilers.

**Methods:**

A total of 400 male Arbor Acres broilers (one-day-old) were randomly assigned to five experimental groups with eight replicates over a 14-day experimental period. Chicks were fed a basal diet (Control group), the basal diet supplemented with 10 g/kg Nat-Pal or 2.5, 5, and 10 g/kg Dis-Pal, respectively.

**Results:**

Dis-Pal linearly increased average body weight and average daily gain, and decreased feed-to-gain ratio of broilers from days 1 to 14, with the 5–10 g/kg Dis-Pal showing the most pronounced effects (p<0.05). An addition of Dis-Pal linearly increased glucose level, and decreased triglyceride level in serum (p<0.05). Compared to the control group, 5 g/kg Dis-Pal increased the digestibility of crude protein and dry matter, enhanced trypsin, lipase, and amylase activities in jejunal digesta, and elevated the ratio between villus height and crypt depth in jejunum and ileum (p<0.05). Dis-Pal linearly increased ileal mucosal glutathione and total antioxidant capacity levels, as well as total superoxide dismutase and catalase activities, with 10 g/kg Dis-Pal addition showing the best effects (p<0.05). The 2.5–10 g/kg Dis-Pal decreased the interferon-γ and tumor necrosis factor-α levels in ileal mucosa compared to the control group (p<0.05). Moreover, Dis-Pal supplementation linearly increased interleukin-10, secretory immunoglobulin A, and immunoglobulin M levels, and decreased the interferon-γ level, quadratically increased total superoxide dismutase and catalase activities in jejunal mucosa (p<0.05).

**Conclusion:**

Dietary Dis-Pal supplementation could improve growth performance, intestinal health, and enhance the nutrient digestibility of young broilers. Supplementation with Dis-Pal was more advantageous for broilers than with Nat-Pal, and its optimal dosage was 5 g/kg.

## INTRODUCTION

The initial growth stage of broiler chicks is a crucial period for their development. During this stage, they exhibit uneven growth, higher mortality rates, and rapid development of their digestive systems in terms of both size and function, which may ultimately affect their later growth performance [1,2]. In responses to these challenges, early nutritional interventions have been suggested and put into practice to improve growth performance.

Palygorskite (Pal) is a naturally occurring hydrated magnesium aluminum silicate [Si_8_Mg_8_O_20_(OH)_2_(H_2_O)_4_·4H_2_O] clay nanomineral with a diameter of 20–40 nm and a length of 0.5–5 μm [3,4]. Its unique crystal structure, stacking mode, and nanoscale dimensions of the rod crystals of Pal give it colloidal, adsorptive, and stability properties [5,6]. In animal nutrition, Pal is used as both a feed additive and a raw feed material for farm animals. Several studies have been performed on broilers, which have shown that the addition of natural Pal (Nat-Pal) has positive effects on growth performance, intestinal health, immune function, and digestive capacity [7–10]. These findings underscore the potential of Nat-Pal to enhance nutrition and health in broiler chicks.

However, the presence of electrostatic and van der Waals forces among the rod crystals in Nat-Pal often leads to the formation of crystal bundles or bulk aggregates, which hinders their potential as a nanomaterial and limits the utilization of their functional characteristics [11,12]. Through various treatments aimed at disaggregating the crystal bundles, the resulting crystal bundles disaggregated palygorskite (Dis-Pal) exhibits significantly improved colloidal, adsorptive, and surface properties compared to Nat-Pal [13,14]. In recent years, advances in the technology for disaggregating Pal crystal bundles have expanded its applications to fields such as biomedicine, membrane separation, catalysis, and other emerging areas, opening up new possibilities for the high-performance utilization of Dis-Pal and marking a significant advancement in its functional capabilities [15].

Based on the aforementioned research reports, it has been determined that the optimal dosage of Nat-Pal in broilers’ diets is 10 g/kg, regardless of whether the diet is in pellet or mash form. However, there is a lack of studies investigating the application of Dis-Pal in early-stage nutrition for broilers. Therefore, this study aims to address this gap by comparing the effects of Nat-Pal and Dis-Pal supplementation. The comparison will evaluate various factors, such as growth performance, organ development, serum biochemical parameters, antioxidant status, apparent nutrient utilization, digestive enzyme activity, and the morphology of the intestinal mucosa in broilers at an early age.

## MATERIALS AND METHODS

### Materials

The Nat-Pal sample was originally sourced from a deposit in the Baihushan Mine, located in Xuyi country, Jiangsu province, P.R. China. The Dis-Pal sample was obtained using ultrasonic-assisted Nat-Pal crystal bundle dissociation technology. The main chemical components of Pal samples were measured by Epsilon 3 X-ray fluorescence spectrometer (Malvern Panalytical). The surface area zeta potential of Pal samples was measured by Accelerated Surface Area and Porosity 2020-M analyzer (Micromeritics Instrument) and ZS90-2000 zeta potential analyzer (Malvern Instrument), respectively. The physicochemical property and chemical composition of Pal samples were shown in Supplemental 1. Both Pal minerals (sieved through a 180-mesh sieve) used in this study were kindly provided by Jinhan New Materials and were used without any additional purification.

### Experimental design, birds, and diets

A total of four hundred male Arbor Acres broiler chickens, aged one-day-old and weighted an average of 44.33±0.14 g, were purchased from a local commercial hatchery. The chicks were randomly assigned to one of five experimental groups with eight replicates of ten birds per replicate. They were raised in 4-level stainless steel cages (120 cm×80 cm×45 cm) in a temperature-controlled room for an experimental period of fourteen-day period. Chicks were fed a basal diet (Control group), the basal diet supplemented with 10 g/kg Nat-Pal, and the basal diet supplemented with 2.5, 5, and 10 g/kg Dis-Pal, respectively. The basic diet (Table 1) and breeding from days 1 to 14 were prepared according to the National Research Council [16] requirements and the AA Broiler Management Guide. Throughout the study, all birds had *ad libitum* access to feed and water.

### Growth performance

Birds’ weight (after a 12-h empty stomach period) and feed consumption in each pen were weighed individually on day 14. Meanwhile, average body weight (ABW), average daily gain (ADG), average daily feed intake (ADFI), and feed-to-gain ratio (F/G) were calculated during the experimental period (days 1 to 14).

### Sample collection

A total of forty broilers (one bird per replicate) with a similar ABW were selected from five groups on day 14 and weight of each chicken recorded. Then, 5 mL blood was collected from the subwing vein using a vacuum coagulation tubelet, and centrifuged at 1,183 ×g for 10 minutes at 4°C to remove impurities. The supernatant was transferred into a clean centrifuge tube and stored at −20°C until analysis. After blood sampling, the birds were slaughtered by cervical dislocation. The liver, thymus, spleen, bursa of Fabricius, gizzard, proventriculus, and pancreas were collected from the euthanized birds and weighed to determine the relative organ weight using the following formula: relative organ weight (g/kg) = organ weight/body weight. The abdominal cavity was opened rapidly, approximately 2 cm segments of the middle portion of the jejunum and ileum were immediately collected, opened longitudinally, flushed with ice-cold 0.9% physiological saline, and stored in 4% paraformaldehyde fix solution (P1110; Solarbio Science & Technology) for subsequent gut histological measurement. The remaining portions of the jejunal and ileal segments mentioned above were used to scrape intestinal mucosa samples using glass microscope slides and immediately frozen in liquid nitrogen until further analysis.

### Preparation of intestinal digesta and mucosal homogenate

The jejunal and ileal digesta and mucosa samples (approximately 0.3 g) were homogenized in a 1:9 ratio of mass to volume with ice-cold 0.90% sodium chloride solution, and homogenized using an Ultra-Turrax homogenizer (Tekmar) until no tissue particles were visible in the solution. The homogenized samples were then centrifuged at 603 ×g for 10 minutes at 4°C to collect the supernatant, which was stored at −20°C for further related parameter analysis. The total protein (TP, cat. A045-4) level was quantified using a corresponding colorimetric assay kit (Jiancheng Bioengineering Institute). Finally, the results of jejunal and ileal digesta and mucosa were corrected by TP concentration.

### Serum biochemical parameters

The serum biochemical parameters, which included TP, albumin (ALB, cat. A028-2), total cholesterol (T-CHO, cat. A111-1), triglyceride (TG, cat. A110-1), glucose (GLU, cat. A154-1) concentrations, alanine aminotransferase (ALT, cat. C009-2) and aspartate aminotransferase (AST, cat. C010-2) activities, were determined using commercial kits from Jiancheng Bioengineering Institute. The manufacturer’s instructions were followed for testing process. The globulin (GLB) content was calculated by subtracting the ALB level from the TP level.

### Apparent utilization rate of nutrients

On days 14 to 16, forty birds (one bird from each pen) with similar weights were placed into individual cages for feeding and used for metabolic experiments. The trays were placed under each cage, and a 3-day total collection of excreta was conducted. Before the collection of excreta, the birds were fasted for 24 hours with free access to water and then fed an equal number of experimental diets at once. The excreta (feathers and feed were removed) were collected from each pen twice per day (at 09:00 and 16:00) and frozen at −20°C. At the end of the 2 collection days, feed intakes were recorded and the excreta from each cage were mixed. The diets and mixture excreta samples were taken and dried to a constant weight in a hot air oven at 105°C for 48 hours, and then cooled to room temperature for 24 hours. The samples were then ground and sieved through a 0.45-mm screen. Samples of the diets and excreta were analyzed for crude protein (CP, 920.39), ether extract (EE, 920.39), dry matter (DM, 934.01), and crude ash (942.05) according to the standard method [17]. The organic matter (OM) content is determined by subtracting the DM and crude ash. The apparent utilization of CP, EE, OM, and DM was calculated using the following formula: apparent nutrient utilization (%) = (Diet [Nutrient× DM]−Excreta [Nutrient×DM])/(Diet [Nutrient×DM])×100.

### Histomorphology structure analysis of jejunum and ileum

The jejunal and ileal segments for morphology were embedded in paraffin after dehydration (n = 8/group). The embedded tissues were then sectioned at 5 μm and then stained with hematoxylin and eosin. Images of the jejunal and ileal tissue were captured using a light microscope (Nikon). The intestinal villus height (VH) and crypt depth (CD) were measured and analyzed for six well-oriented villus or crypts for each paraffin section using the Image J software (NIH Image J System). Then, the ratio of villus height to crypt depth (V/C) was then calculated.

### Digestive enzyme activity assay

The activities of trypsin (TRS, cat. A080-2), lipase (LPS, cat. A054-2), and amylase (AMS, cat. A016-1) in the jejunal and ileal digesta were measured using spectrophotometry and commercial kits from Jiancheng Bioengineering Institute.

### Intestinal mucosal antioxidant capacity assay

The concentrations of malondialdehyde (MDA, cat. A003-1), glutathione (GSH, cat. A006-1), and total antioxidant capacity (T-AOC, cat. A015-2), and the activities of total superoxide dismutase (T-SOD, cat. A001-1), glutathione peroxidase (GSH-PX, cat. A005-1), and catalase (CAT, cat. A007-1) in the jejunal and ileal mucosa were assayed using commercial kits (Jiancheng Bioengineering Institute).

### Intestinal mucosal immune indices measure

The enzyme-linked immunosorbent assay was performed to examine the levels of cytokines and immunoglobulins in both jejunal and ileal mucosa following the protocols of instructions. The quantitation kits were purchased from Nanjing Hongsheng, including interleukin-1β (IL-1β, cat. CK-E60036), interleukin-10 (IL-10, cat. CK-E60031), interferon-γ (IFN-γ, cat. CK-E60027), tumor necrosis factor-α (TNF-α, cat. CK-E60161), secretory immunoglobulin A (sIgA, cat. CK-E60068), immunoglobulin G (IgG, cat. CK-E60107), and immunoglobulin M (IgM, cat. CK-E60110). The specific steps of the experimental operation were previously described by Du et al [18].

### Statistical analysis

Data were analyzed by one-way analysis of variance (ANOVA) in SPSS statistical software for Windows (ver. 22.0, IBM), and Duncan’s multiple comparisons were examined to separate the difference among treatments. Orthogonal polynomial contrasts were also employed to test the linear and quadratic effects of the different levels of Dis-Pal. An individual bird from each pen served as the experimental unit for data analysis. A p-value less than 0.05 was considered significant, and the results were presented as means with their corresponding pooled standard errors.

## RESULTS

### Growth performance

Dietary Dis-Pal administration linearly increased the ABW and ADG, and decreased the F/G of broilers (Table 2, p<0.05). Compared to the control group, the addition of 5 and 10 g/kg Dis-Pal to the diet enhanced the ABW and ADG, and reduced the F/G in broilers (p<0.05). There were no differences between the Nat-Pal and Dis-Pal treated groups. In contrast, dietary 10 g/kg Nat-Pal supplementation did not have any effect on ABW, ADFI, ADG, or F/G when compared with the control group. Additionally, there were no differences in the ADFI among all treatments.

### Relative organ weight

Neither Nat-Pal nor Dis-Pal had any effect on the relative weight of the liver, thymus spleen, bursae of Fabricius, gizzard, proventriculus or pancreas of broilers (Table 3).

### Serum biochemistry

The supplementation of Dis-Pal linearly reduced the TG level and increased the GLU level in serum (Table 4, p<0.05). The addition of 10 g/kg Dis-Pal increased the level of serum GLU when compared with other groups (p<0.05). However, dietary Nat-Pal and Dis-Pal addition did not affect the TP, ALB, GLB, TC, TG, ALT, and AST levels in serum of broilers.

### Apparent nutrient utilization

As exhibited in Table 5, dietary supplemental Dis-Pal had a linear increase on the CP digestibility and quadratic increase in the DM digestibility of broilers (p<0.05). Specifically, the DM digestibility was higher in the 2.5–5 g/kg Dis-Pal addition groups compared to the control and 10 g/kg Nat-Pal groups (p<0.05). Compared to the control and 10 g/kg Nat-Pal groups, dietary 5 g/kg Dis-Pal supplementation resulted in a higher digestibility of CP of broilers (p<0.05). In addition, while there were no differences in the digestibility of EE and OM of broilers between the control group and the groups supplemented with Pal, there was a trend towards increased digestibility with Pal supplementation.

### Intestinal morphology

In Table 6, it can be seen that dietary supplementation with Dis-Pal not only linearly increased the VH and the VH/CD in the jejunum, but also increased the VH/CD in the ileum of broilers (p<0.05). Compared to the control and 2.5 g/kg Dis-Pal groups, supplementing with 5 and 10 g/kg Dis-Pal resulted in increases in VH, and VH/CD in the jejunum (p<0.05). Additionally, supplementing with 10 g/kg Nat-Pal in the diet increased VH/CD in the jejunum of broilers (p<0.05) compared to the control group and 2.5 g/kg Dis-Pal groups. In the jejunum and ileum, dietary administration of 5 g/kg Dis-Pal increased the VH/CD compared to the 2.5 g/kg Dis-Pal group (p<0.05). Moreover, 5 g/kg Dis-Pal increased the VH/CD in the ileum compared to the control group (p<0.05). However, there was no difference in the CD among all treatments in the intestine of broilers.

### Digestive enzymes activity

Compared to the control group, dietary Dis-Pal supplementation linearly increased the TRS and LPS activities, and quadratically increased the AMS activity in the jejunal digesta of broilers (Table 7, p<0.05). Specifically, in the jejunal digesta, compared to the control group, the addition of 2.5–10 g/kg Dis-Pal and 10 g/kg Nat-Pal led to an increase in TRS activity, while 5–10 g/kg Dis-Pal resulted in an increase in LPS activity, and 2.5–10 g/kg Dis-Pal led to an increase in AMS activity (p<0.05). However, there were no changes in intestinal digesta digestive enzyme activities among the different doses of the Dis-Pal group.

### Antioxidant capacity of intestinal mucosa

The antioxidant capacity of the intestinal mucosa in broilers is shown in Table 8. When a basal diet was supplemented with Dis-Pal, there was a linear increase in the levels of GSH and T-AOC, as well as the activities of T-SOD and CAT in the ileum. Additionally, there was a quadratic increase in the activities of T-SOD and CAT in the jejunum (p<0.05). A 10 g/kg supplementation of Dis-Pal also increased the levels of GSH and T-AOC, as well as the activity of CAT in the ileum of broilers (p<0.05). Furthermore, there was no difference in the jejunal mucosal antioxidant index among broilers fed a basal diet supplemented with Nat-Pal and Dis-Pal.

### Immune indices of intestinal mucosa

As presented in Table 9, dietary supplemental Dis-Pal had a linear increase in the concentrations of IL-10, sIgA, and IgM in the jejunal mucosa, as well as in the concentration of IgM in the ileal mucosa (p<0.05). There was also a linear decrease in the level of IFN-γ in the jejunal mucosa and the levels of IFN-γ and TNF-α in the ileal mucosa, with a quadratic decrease in the IFN-γ level in the ileal mucosa of broilers (p<0.05). Specifically, the administration of 10 g/kg Dis-Pal resulted in a decrease in IFN-γ level compared to the control and 2.5 g/kg Dis-Pal groups (p<0.05). An incorporation of 10 g/kg Nat-Pal and 5–10 g/kg Dis-Pal to the diet increased the sIgA concentration in the jejunal mucosa compared to the control group (p<0.05). Additionally, dietary supplementation with 2.5–10 g/kg Dis-Pal and 10 g/kg Nat-Pal led to a decrease in IFN-γ and TNF-α concentrations in the ileal mucosa compared to the control group (p<0.05). However, Pal treatment did not have any effect on the levels of IL-1β, IL-10, TNF-α, IgG, and IgM in the jejunal mucosa, or on the levels of IL-1β, IL-10, sIgA, IgG, and IgM in the ileal mucosa of broilers.

## DISCUSSION

The positive consequences of incorporating Nat-Pal into broiler diets have been welldocumented in previous studies. Chen et al [9,19] found that supplementing diets with Nat-Pal (5 g/kg and 10 g/kg) did not affect the growth performance of broilers during the first 21 days. Likewise, no changes in growth performance were observed when Nat-Pal was added at 5 or 10 g/kg by day 42, although a higher supplementation level of 20 g/kg increased the F/G [20]. Consistent with these findings, our study also found that adding 10 g/kg of Nat-Pal to the diet did not affect broiler growth performance. However, another study reported that dietary supplementation with Nat-Pal (0, 5, 10, 15, and 20 g/kg) led to both linear and quadratic increases in ADG and ADFI of birds during the starter period [7]. Additionally, feeding broilers with 10 g/kg of ultra-fine ground Pal significantly decreased the F/G from days 1 to 42 compared to feeding the basal diet, but there was no significant difference compared to adding 10 g/kg Nat-Pal to the basal diet [10]. Similarly, our study found that adding Dis-Pal (5 or 10 g/kg) to the diet significantly improved the growth performance indicators (ABW, ADG, and F/G) of broilers compared to the control group, but there was no significant difference compared to adding 10 g/kg Nat-Pal. Although the exact mechanism of how Pal affects the growth performance of broilers is still unclear, the different results may be related to the type, purity, particle size, and level of supplementation. The potential mechanisms through which Dis-Pal enhances broiler growth can be summarized as follows: the newly developed Dis-Pal nanomaterials exhibit superior properties—such as a larger specific surface area, enhanced adsorption performance, and faster release of elements—compared to Nat-Pal, following the disaggregation of Pal crystal bundles. These properties enable Dis-Pal to exert a more pronounced effect on feed efficiency and overall broiler growth.

Relative organ weight is considered to be an indicator of organ growth and development. The weight of the gizzard and proventriculus can serve as markers for the development of the digestive system, while the weights of the thymus, spleen, and bursae of Fabricius can indicate the development of the immune system [21]. Moreover, the liver and pancreas are crucial digestive organs that secrete digestive enzymes and other digestive juices [10]. However, dietary supplementation with 5 and 10 g/kg Nat-Pal may have a negative impact on the relative weight of the pancreas on day 42, but does not seem to affect the weight of the pancreas on day 21 [19]. In terms of internal organs, adding 10 g/kg of nanoclay minerals to the broilers’ diet once a week can decrease the weight of the gizzard, proventriculus, spleen, and bursa of Fabricius, while increasing the weight of the liver and pancreas on day 28 [22]. Our study found that adding 5 and 10 g/kg of Dis-Pal led to an increase in pancreatic weight. This could be due to the promotion of pancreatic digestive function and the secretion of related enzymes by Pal, which may explain the enhanced activity of digestive enzymes in the intestinal mucosa. However, our findings do not entirely align with previous studies, which may be attributed to differences in the physical, chemical, and biological properties of the mineral materials, as well as the age and feeding environment of the broilers.

Blood biochemical parameters are useful for assessing the health and metabolic status of broilers, as well as their response to environmental factors [23]. The activity levels of ALT and AST are considered indicators of liver damage, while the concentration of proteins (TP and ALB) reflects the functional status of hepatocytes [24]. Additionally, the serum concentrations of T-CHO and TG are considered indicators of lipid metabolism [25]. GLU is the main product of the final digestion and decomposition of carbohydrates in the digestive tract of broiler chickens. Therefore, serum glucose levels can reflect the body’s efficiency in digesting and absorbing feed carbohydrates. In this study, the addition of 10 g/kg of Dis-Pal significantly increased serum GLU levels, likely due to the observed rise in ADFI, which enhanced carbohydrate digestion and absorption. Moreover, neither Nat-Pal nor Dis-Pal supplementation significantly affected other serum biochemical parameters, indicating that Pal additives do not adversely affect broiler growth. These findings align with previous studies [9,10].

Previous research has shown that incorporating silicate minerals into broiler diets can improve growth performance by increasing the digestibility of energy and protein [26]. In a study by Zhou et al [8], supplementation with a combination of zeolite and Pal was found to enhance the apparent digestibility of CP and gross energy in the jejunal mucosa of broilers. Similarly, Du et al [10] reported that including 10 g/kg Nat-Pal in the diet did not affect the apparent utilization of nutrients (OM, EE, and CP) of broilers at days 17 to 19, which is consistent with our findings. Our study also showed that dietary supplementation with Pal increased the utilization of measured nutrients, with the addition of 5 g/kg Dis-Pal having a stronger effect than 10 g/kg Nat-Pal. This can be attributed to the gel-forming properties of clay minerals in the intestines, which prolong the residence time of digesta and improve the effectiveness of endogenous enzymes in digesting fats, proteins, and carbohydrates. The higher specific surface area and colloid performance of Dis-Pal compared to Nat-Pal may explain its superior effect on digesta viscosity and passage rate through the small intestine. Therefore, the inclusion of Dis-Pal in broiler diets is more beneficial than Nat-Pal.

Intestinal mucosal micromorphological features, including VH, CD, and VH/CD, play a critical role in maintaining intestinal health [27]. In this study, the inclusion of Pal in the diet increased VH and VH/CD in the jejunum and ileum of broilers. Interestingly, we found that adding 5 g/kg Dis-Pal had a greater effect on promoting intestinal development than 10 g/kg Nat-Pal. This result is consistent with previous studies [9,18,28]. Incorporating clay minerals has been shown to effectively improve cellular redox status and combat oxidative damage. Dietary Nat-Pal supplementation can increase the antioxidant ability of the jejunum [18]. The protective effect of Pal on intestinal integrity and barrier function may stem from its unique structural characteristics, which enable it to adsorb onto the intestinal mucosa surface, forming a physical barrier against harmful substances such as toxins, bacteria, and viruses [29]. Additionally, Pal has been shown to enhance intestinal health by increasing the renewal rate of villous cells, stimulating mucus secretion, and improving immune function [30–33].

The growth and production of poultry are primarily dependent on the digestion and absorption of nutrients in the small intestine [34]. The pancreas secretes a range of digestive enzymes into the small intestine that are essential for breaking down nutrients [35]. Therefore, this study evaluated the effect of Pal on digestion and absorption in the intestines by measuring the activities of enzymes (TRS, AMS, and LPS) in the small intestine. According to Wu et al [36], adding clinoptilolite to broiler diets significantly increased the activities of digestive enzymes, including protease, chymotrypsin, TRS, and AMS, in the small intestinal contents and promoted gut development at an early age. Similarly, broilers fed diets supplemented with a combination of zeolite and Pal showed significantly higher activities of AMS, LPS, and TRS in jejunal digesta [8]. The results of this study also showed that 5 or 10 g/kg dietary Nat-Pal supplementation significantly enhanced the activities of digestive enzymes in jejunal digesta compared to the control group. This enhancement may be attributed to the promotion of pancreas and intestinal mucosal development by Pal, as measured in this study.

The redox system in animals plays a crucial role in regulating various biological functions [37]. Studies have shown that clay minerals can effectively improve cellular redox status, maintain redox balance, and counteract oxidative damage in the body [38,39]. Wu et al [40] also found that adding 2% zeolite to feed increased T-SOD and CAT activity in broiler liver, while reducing MDA levels. Chen et al found that adding 10 g/kg of attapulgite significantly increased T-SOD activity in the jejunum and ileum mucosa of broiler chickens [9]. Similarly, adding 10 g/kg of low viscosity attapulgite to feed increased serum SOD activity and jejunal T-AOC levels in broiler chickens, while reducing serum and jejunal MDA levels [18]. *In vitro* cell experiments have also shown that fibrous clay minerals can effectively inhibit the generation of lipid peroxides, possibly due to the large number of adsorption sites on their surface reacting with hydroxyl radicals to exert antioxidant capacity [41]. In this experiment, the addition of non-attapulgite to feed increased the antioxidant activity of duodenal and jejunal mucosa to varying degrees, with Dis-Pal showing a better effect than Nat-Pal. This may be due to the more dispersed rod crystal structure, larger specific surface area, and stronger surface-active groups of attapulgite dissociated from rod crystal bundles.

Clay minerals are commonly used in animal feed as immunostimulants to enhance immune function and disease resistance [42]. The overproduction of pro-inflammatory cytokines such as IL-1β, TNF-α, and IFN-γ can lead to the endocytosis of intestinal tight junction proteins, resulting in compromised intestinal integrity [43]. On the other hand, IL-10, an important anti-inflammatory cytokine, plays a crucial role in preventing excessive immune response and tissue damage by regulating the production of pro-inflammatory cytokines [44]. In a study by Du et al [18], the incorporation of 10 g/kg Nat-Pal into the diet reduced the levels of IL-1β and IFN-γ in both the serum and jejunal mucosa of broilers. Similarly, Zha et al [28] found that supplementing broiler diets with 250–2,000 mg/kg of a Pal-based antibacterial agent resulted in a linear and/or quadratic decrease in the levels of pro-inflammatory cytokines (IL-1β, TNF-α, and IFN-γ) in the jejunal and ileal mucosa of broilers. In our study, we observed a decrease in TNF-α and IFN-γ levels in the ileal mucosa of broilers treated with Pal. This finding is consistent with the results of Chen et al [45], who reported that Pal administration down-regulated the mRNA expression levels of intestinal pro-inflammatory cytokines in the intestines of broilers, thereby inhibiting systemic inflammatory responses induced by lipopolysaccharide at an early age. Pal, a traditional fibrous aluminosilicate mineral, has been shown to possess anti-inflammatory properties by inhibiting neutrophil migration, altering leukocyte cell infiltration at inflammation sites, and restricting myeloperoxidase activity, as well as reducing the expression of pro-inflammatory cytokines in a murine inflammation model [46,47]. Immunoglobulins are commonly used to assess the immune status of animals due to their important roles involved in immune function. Chen et al [9] reported that supplementing broiler diets with 10 g/kg Nat-Pal significantly elevated the levels of ileal sIgA and IgM, and showed a tendency to increase jejunal sIgA, IgG, and IgM, as well as ileal IgG contents, resulting in improved mucosal immunity of broilers. Similarly, Wang et al [48] found that supplementing with 10 g/kg Nat-Pal increased the intestinal mucosal levels of sIgA, IgG, and IgM to varying degrees, thereby enhancing intestinal immune function in ducks. In the current study, we also observed an increase in intestinal sIgA, IgG, and IgM levels with the addition of Pal to the diet, regardless of type and dosage. This increase in intestinal mucosal immunoglobulin levels may be attributed to the key chemical composition of Pal, aluminosilicate, which has been shown to possess immune regulatory activities, promoting antibody production and improving immune organ development and function [49]. Additionally, Zhang et al [50] reported that supplementing Nat-Pal may enhance the intestinal immune system by increasing the number of intraepithelial lymphocytes in the intestinal epithelium. These findings further support the role of Pal in enhancing intestinal immune function and protecting intestinal health.

## CONCLUSION

In summary, our study demonstrated that supplementing broiler feed with Pal can improve growth performance, protect intestinal health, and enhance nutrient digestibility of young broilers. Notably, Dis-Pal proved more effective than Nat-Pal, with 5 g/kg identified as the optimal supplementation level for young broiler diets.

## Figures and Tables

**Table 1:** Compositions and nutrient levels of the experimental basal diet (as-fed basis, %)

Ingredients	Contents
Corn	58.00
Soybean meal	30.00
Corn gluten meal	4.00
Soybean oil	3.00
Dicalcium phosphate	2.00
Limestone	1.20
*L*-Lysine	0.30
*DL*-Methionine	0.20
Sodium chloride	0.30
Mineral-vitamin premix^1)^	1.00
Total	100.00
Calculated nutrient levels	
Metabolizable energy (MJ/kg)	12.78
Crude protein	21.11
Calcium	0.99
Total phosphorus	0.69
Available phosphorus	0.46
Lysine	1.15
Methionine	0.55
Methionine+cystine	0.90
Analyzed nutrient levels	
Crude protein	20.97
Calcium	0.95
Total phosphorus	0.70

1) The mineral-vitamin premix provided the followings per kilogram of diets: vitamin A, 12,000 IU; vitamin D_3_, 3,000 IU; vitamin E, 30 IU; menadione, 1.3 mg; thiamin, 2.2 mg; riboflavin, 8 mg; nicotinamide, 40 mg; choline chloride, 600 mg; calcium pantothenate, 10 mg; pyridoxine·HCl, 4 mg; biotin, 0.04 mg; folic acid, 1 mg; vitamin B_12_, 0.013 mg; Fe, 80 mg; Cu, 8.0 mg; Mn, 110 mg; Zn, 65 mg; I, 1.1 mg; Se, 0.3 mg.

**Table 2:** Effects of different supplemental levels of palygorskite on the growth performance of broilers at 14 days of age

Items	Control	10 g/kg Nat-Pal	Dis-Pal level (g/kg)			SEM	p-value		
	
			2.5	5	10		Treatment	Linear	Quadratic
ABW (g)	410.38^b^	429.00^ab^	424.38^ab^	441.50^a^	441.00^a^	3.50	0.019	0.003	0.350
ADFI (g)	32.60	32.99	32.70	32.92	33.39	0.29	0.931	0.436	0.805
ADG (g)	26.16^b^	27.47^ab^	27.17^ab^	28.37^a^	28.31^a^	0.25	0.020	0.004	0.337
F/G	1.25^a^	1.20^ab^	1.21^ab^	1.16^b^	1.18^b^	0.01	0.042	0.012	0.146

a,b Means within a row with different superscripts are different at p<0.05.

Nat-Pal, natural palygorskite; Dis-Pal, disaggregation crystal bundles of palygorskite; SEM, standard error of the mean; ABW, average body weight; ADFI, average daily feed intake; ADG, average daily weight gain; F/G, feed to gain ratio.

**Table 3:** Effects of different supplemental levels of palygorskite on relative organ weight of broilers at 14 days of age (g/kg)

Items	Control	10 g/kg Nat-Pal	Dis-Pal level (g/kg)			SEM	p-value		
	
			2.5	5	10		Treatment	Linear	Quadratic
Liver	32.99	31.93	30.85	30.93	31.63	0.58	0.791	0.524	0.313
Thymus	4.71	4.61	4.64	4.59	4.63	0.18	1.000	0.892	0.903
Spleen	0.81	0.76	0.78	0.73	0.81	0.03	0.926	0.917	0.432
Bursae of Fabricius	2.52	2.32	2.19	2.26	2.37	0.09	0.821	0.684	0.278
Gizzard	22.33	21.66	23.23	22.00	21.65	0.39	0.716	0.429	0.500
Proventriculus	7.57	7.75	7.56	7.69	7.63	0.12	0.987	0.811	0.934
Pancreas	4.75	4.77	4.64	4.82	4.81	0.12	0.991	0.780	0.845

Nat-Pal, natural palygorskite; Dis-Pal, disaggregation crystal bundles of palygorskite; SEM, standard error of the mean.

**Table 4:** Effects of different supplemental levels of palygorskite on serum biochemical indices of broilers at 14 days of age

Items	Control	10 g/kg Nat-Pal	Dis-Pal level (g/kg)			SEM	p-value		
	
			2.5	5	10		Treatment	Linear	Quadratic
TP (g/L)	22.92	23.17	23.12	23.73	23.93	0.21	0.508	0.097	0.995
ALB (g/L)	15.65	15.26	15.45	15.10	15.11	0.28	0.973	0.538	0.878
GLB (g/L)	7.28	7.91	7.67	8.63	8.82	0.40	0.734	0.192	0.909
T-CHO (mmol/L)	4.55	4.36	4.47	4.40	4.35	0.09	0.962	0.533	0.932
TG (mmol/L)	0.62	0.55	0.56	0.55	0.50	0.01	0.150	0.024	0.884
GLU (mmol/L)	12.23^b^	13.01^b^	12.59^b^	13.06^b^	14.02^a^	0.15	0.001	<0.001	0.273
ALT (U/L)	1.38	1.38	1.34	1.33	1.38	0.04	0.995	0.708	0.982
AST (U/L)	27.25	25.32	25.79	25.33	25.16	0.54	0.744	0.380	0.587

a,b Means within a row with different superscripts are different at p<0.05.

Nat-Pal, natural palygorskite; Dis-Pal, disaggregation crystal bundles of palygorskite; SEM, standard error of the mean; TP, total protein; ALB, albumin; GLB, globulin; T-CHO, total cholesterol; TG, triglyceride; GLU, glucose; ALT, alanine aminotransferase; AST, aspartate aminotransferase.

**Table 5:** Effects of different supplemental levels of palygorskite on nutrient apparent digestibility of broilers at 14 days of age (%)

Items	Control	10 g/kg Nat-Pal	Dis-Pal level (g/kg)			SEM	p-value		
	
			2.5	5	10		Treatment	Linear	Quadratic
CP	62.34^c^	62.89^bc^	64.66^abc^	67.88^a^	66.72^ab^	0.66	0.026	0.008	0.182
EE	71.91	72.16	72.66	75.21	72.39	0.85	0.755	0.645	0.356
OM	69.50	71.43	71.05	72.21	71.98	0.92	0.908	0.380	0.683
DM	70.44^c^	70.55^c^	71.32^ab^	71.69^a^	70.98^bc^	0.12	0.001	0.053	0.001

a–c Means within a row with different superscripts are different at p<0.05.

Nat-Pal, natural palygorskite; Dis-Pal, disaggregation crystal bundles of palygorskite; SEM, standard error of the mean; CP, crude protein; EE, ether extract; OM, organic matter; DM, dry matter.

**Table 6:** Effects of different supplemental levels of palygorskite on intestinal morphology of broilers at 14 days of age

Items	Control	10 g/kg Nat-Pal	Dis-Pal level (g/kg)			SEM	p-value		
	
			2.5	5	10		Treatment	Linear	Quadratic
Jejunum									
VH (μm)	779.04^b^	820.13^ab^	790.55^b^	850.31^a^	859.06^a^	8.64	0.004	0.001	0.940
CD (μm)	134.31	128.24	133.70	131.29	128.10	1.42	0.516	0.146	0.684
VH/CD	5.89^b^	6.44^a^	5.95^b^	6.53^a^	6.78^a^	0.08	<0.001	<0.001	0.505
Ileum									
VH (μm)	710.10	751.93	727.16	810.56	747.56	12.21	0.092	0.077	0.104
CD (μm)	152.64	147.05	147.02	145.16	144.96	1.96	0.752	0.231	0.555
VH/CD	4.74^b^	5.18^ab^	5.04^b^	5.67^a^	5.20^ab^	0.10	0.032	0.031	0.059

a,b Means within a row with different superscripts are different at p<0.05.

Nat-Pal, natural palygorskite; Dis-Pal, disaggregation crystal bundles of palygorskite; SEM, standard error of the mean; VH, villus height; CD, crypt depth; VH/CD, the ratio between villus height and crypt depth.

**Table 7:** Effects of different supplemental levels of palygorskite on intestinal digestive enzyme activities of broilers at 14 days of age

Items	Control	10 g/kg Nat-Pal	Dis-Pal level (g/kg)			SEM	p-value		
	
			2.5	5	10		Treatment	Linear	Quadratic
Jejunum									
TRS (U/mg protein)	3,653.70^b^	4,403.29^a^	4,408.75^a^	4,779.54^a^	4,758.38^a^	120.05	0.014	0.003	0.140
LPS (U/g protein)	49.15^b^	58.13^ab^	55.51^ab^	62.22^a^	63.59^a^	1.65	0.035	0.002	0.462
AMS (U/mg protein)	46.17^b^	49.43^ab^	54.55^a^	54.13^a^	51.14^ab^	0.86	0.006	0.066	0.002
Ileum									
TRS (U/mg protein)	3,406.19	3,821.98	3,671.00	3,691.55	3,874.55	114.57	0.752	0.225	0.875
LPS (U/g protein)	42.71	45.07	45.38	49.45	43.95	1.41	0.647	0.584	0.203
AMS (U/mg protein)	55.56	55.79	54.48	57.52	56.52	0.75	0.785	0.439	0.981

Data represent the means of 8 replicates per treatment.

a,b Means within a row with different superscripts are different at p<0.05.

Nat-Pal, natural palygorskite; Dis-Pal, disaggregation crystal bundles of palygorskite; SEM, standard error of the mean; TRS, trypsin; LPS, lipase; AMS, amylase.

**Table 8:** Effects of different supplemental levels of palygorskite on intestinal mucosa antioxidant capacity of broilers at 14 days of age

Items	Control	10 g/kg Nat-Pal	Dis-Pal level (g/kg)			SEM	p-value		
	
			2.5	5	10		Treatment	Linear	Quadratic
Jejunum									
MDA (nmol/mg protein)	0.42	0.40	0.34	0.33	0.32	0.02	0.477	0.060	0.352
GSH (nmol/mg protein)	12.78	13.07	14.13	14.57	14.01	0.35	0.469	0.244	0.229
T-SOD (U/mg protein)	12.36	12.48	14.18	14.35	12.28	0.36	0.159	0.979	0.012
GSH-PX (U/mg protein)	17.13	19.06	19.21	19.82	19.63	0.54	0.546	0.091	0.283
T-AOC (mmol/mg protein)	125.47	128.16	134.51	154.46	138.50	3.70	0.099	0.062	0.077
CAT (U/mg protein)	3.85	4.35	4.21	4.46	4.09	0.09	0.220	0.222	0.042
Ileum									
MDA (nmol/mg protein)	0.39	0.36	0.35	0.34	0.34	0.01	0.493	0.097	0.440
GSH (nmol/mg protein)	9.80^c^	10.51^ab^	10.05^ab^	10.96^b^	12.19^a^	0.20	<0.001	<0.001	0.157
T-SOD (U/mg protein)	13.08	14.13	14.12	14.14	15.01	0.25	0.188	0.030	0.876
GSH-PX (U/mg protein)	15.82	17.46	16.28	17.95	16.57	0.30	0.140	0.186	0.168
T-AOC (mmol/mg protein)	116.87^c^	138.10^ab^	123.72^c^	124.67^bc^	145.07^a^	4.73	0.001	<0.001	0.164
CAT (U/mg protein)	3.68^b^	4.24^ab^	3.68^b^	3.78^b^	4.53^a^	0.10	0.013	0.006	0.075

a–c Means within a row with different superscripts are different at p<0.05.

Nat-Pal, natural palygorskite; Dis-Pal, disaggregation crystal bundles of palygorskite; SEM, standard error of the mean; MDA, malondialdehyde; GSH, glutathione; T-SOD, total superoxide dismutase; GSH-PX, glutathione peroxidase; T-AOC, total antioxidant capacity: CAT, catalase.

**Table 9:** Effects of different supplemental levels of palygorskite on intestinal immune function of broilers at 14 days of age

Items	Control	10 g/kg Nat-Pal	Dis-Pal level (g/kg)			SEM	p-value		
	
			2.5	5	10		Treatment	Linear	Quadratic
Jejunum									
IL-1β (pg/mg protein)	134.93	122.84	126.24	121.69	121.12	2.34	0.322	0.055	0.436
IL-10 (pg/mg protein)	15.14	17.14	16.36	16.98	17.16	0.31	0.187	0.033	0.442
IFN-γ (pg/mg protein)	30.18^a^	26.01^ab^	27.94^a^	25.62^ab^	22.71^b^	0.78	0.027	0.001	0.816
TNF-α (pg/mg protein)	14.71	13.44	13.75	13.46	13.18	0.30	0.561	0.142	0.642
sIgA (ng/mg protein)	418.02^b^	482.17^a^	454.57^ab^	480.47^a^	489.17^a^	8.35	0.033	0.003	0.410
IgG (μg/mg protein)	548.27	585.86	557.6	582.23	594.21	10.63	0.623	0.138	0.956
IgM (μg/mg protein)	164.44	184.22	177.91	182.17	189.00	3.74	0.294	0.049	0.698
Ileum									
IL-1β (pg/mg protein)	145.95	131.03	138.28	130.11	133.42	2.83	0.386	0.112	0.385
IL-10 (pg/mg protein)	15.15	16.61	16.53	16.69	16.59	0.25	0.258	0.053	0.146
IFN-γ (pg/mg protein)	32.65^a^	27.89^b^	28.51^b^	26.48^b^	28.23^b^	0.55	0.003	0.004	0.013
TNF-α (pg/mg protein)	19.89^a^	17.07^b^	17.55^b^	17.08^b^	17.31^b^	0.33	0.023	0.011	0.068
sIgA (ng/mg protein)	472.89	507.94	504.5	511.92	508.22	6.86	0.374	0.121	0.274
IgG (μg/mg protein)	721.17	769.55	749.98	770.25	777.66	9.60	0.348	0.069	0.638
IgM (μg/mg protein)	196.84	212.54	207.08	215.78	217.82	3.10	0.208	0.024	0.548

Data represent the means of 8 replicates per treatment.

a,b Means within a row with different superscripts are different at p<0.05.

Nat-Pal, natural palygorskite; Dis-Pal, disaggregation crystal bundles of palygorskite; SEM, standard error of the mean; IL-1β, interleukin-1β; IL-10, interleukin-10; IFN-γ, interferon-γ; TNF-α, tumor necrosis factor-α; sIgA, secretory immunoglobulin A; IgG, immunoglobulin G; IgM, immunoglobulin M.

## SUPPLEMENTARY MATERIAL

**Supplement 1.** Physicochemical property and chemical composition of palygorskite samples.
