# Supplementary material for: Natural and crystal bundles disaggregated palygorskite in young broilers: a comparison study
Source: Anim Biosci. 2025 Aug 12;39(1):250150. doi: 10.5713/ab.25.0150 (PMC12754458; doi:10.5713/ab.25.0150)
Supplement: Supplementary file 1 [file ab-25-0150-Supplementary-1.pdf]

Supplement 1. Physicochemical property and chemical composition of palygorskite samples

Items[1)]	Nat-Pal	Dis-Pal
BET surface area (m^2/g)	163.28	183.11
Zeta potential (mV)	-17.81	-20.47
SiO_2 (%)	62.83	60.91
Al_2O_3 (%)	10.53	11.84
Fe_2O_3 (%)	9.63	9.84
MgO (%)	7.92	7.79
CaO (%)	3.61	4.22
K_2O (%)	1.89	2.08
TiO_2 (%)	1.31	1.31
P_2O_5 (%)	1.31	1.15

[1)] Nat-Pal, natural palygorskite; Dis-Pal, disaggregation crystal bundles of palygorskite.
